# Do Your School Mates Influence How Long You Game? Evidence from the U.S.

**DOI:** 10.1371/journal.pone.0160664

**Published:** 2016-08-05

**Authors:** Aliaksandr Amialchuk, Ales Kotalik

**Affiliations:** 1 Department of Economics, University of Toledo, Toledo, Ohio, United States of America; 2 Division of Biostatistics, School of Public Health, University of Minnesota, Minneapolis, Minnesota, United States of America; Hospital Universitari de Bellvitge, SPAIN

## Abstract

The goal of this paper is to estimate peer influence in video gaming time among adolescents. Using a nationally representative sample of the U.S. school-aged adolescents in 2009–2010, we estimate a structural model that accounts for the potential biases in the estimate of the peer effect. Our peer group is exogenously assigned and includes one year older adolescents in the same school grade as the respondent. The peer measure is based on peers’ own reports of video gaming time. We find that an additional one hour of playing video games per week by older grade-mates results in .47 hours increase in video gaming time by male responders. We do not find significant peer effect among female responders. Effective policies aimed at influencing the time that adolescents spend video gaming should take these findings into account.

## Introduction

Global sales and popularity of video games have been rising steadily in the recent decade and are expected to continue their growth in the foreseeable future [1]. Players under the age of 18 account for about one-quarter of the audience of this growing industry and as the games become increasingly realistic, there has been growing concern among parents, the media and the research community about important social and behavioral effects of video gaming on adolescents [2,3].

The existing literature suggests significant social, behavioral and clinical effects of video gaming. Some of the literature has linked violent video games to aggressive and antisocial behaviors among children and adolescents. Early literature suggests that excessive arcade video gaming may develop into a behavior which resembles a gambling addiction [4]. It was found that children who play more video games are more likely to behave violently and get into arguments [5]. This relationship was confirmed in another study of 8^th^ and 9^th^ graders in the U.S. Midwestern schools [6] and later in meta-analysis [5] found this relationship to exist in different cultures. Another study [7] found the opposite to be true for pro-social video games. Some studies investigated the link to school performance and attention and found a negative effect of video games on school performance [6,8] and college engagement [9]. However another study [10] found that video gaming had no significant relationship to poor academic performance after controlling for other risk factors. It was found that video gaming among girls was associated with higher odds of getting into a serious fight and carrying a weapon to school [3]. Among other studies, [11] found a link between video gaming and sleep disorders and hyperactivity, [12] found a link to attention deficits and [13] using longitudinal data found some evidence of relationship between gaming at age 8 or 9 and behavioral and psychological problems measured at age 15. Yet, in a recent experiment on adults with and without autism [14], there was found no effect of violent-video-game exposure on aggressive behavior in either group. Moreover, in a recent meta-analysis, It was concluded [15] that video games have little influence on several children’s and adolescents’ behaviors, including aggression, mental health, prosocial behavior, and academic performance. Also, it was noted that women are often misrepresented in video games [16], however there was no relationship found between video game use and sexist attitudes towards women using a 3 year longitudinal study of adolescent students in Germany [16]. Other literature has documented several positive effects of video gaming. It was found that video gaming was associated with lower odds of smoking in boys and lower odds of reporting depression in girls [3]; and gaming was not found to be associated with alcohol, drug or caffeine consumption or body-mass index [3]. In a longitudinal study of students in Canada over 4 years [17], it was found that playing sports video games is associated with participating in active sports more. In addition, experienced gamers have been found to have superior visual, spatial and attention skills [18,19,20]. Consistent with this strand of literature, a recent study using functional MRI scanning of the participant gamers [21], found that violent video games result in aggression desensitization and improvements in spatial attention. Moreover, several successful health interventions among children and adolescents used video-game format [22]. The link between video gaming and these important outcomes warrants examination of the factors that might change video gaming behavior of adolescents.

In this paper, we focus on estimating peer effects in the time spent video gaming by adolescents in the U.S. The literature hypothesized that the social network and peer influence play an important role in video gaming behavior of adolescents. Support to this hypothesis is lent by survey data, which indicated that video gaming often happens in group setting—with friends or family members [23,24]. If peer effects in video gaming activities exist, such peer effects “may serve to amplify the effects of interventions” targeted at changing behavior of individual gamers [25].

Most of the previous research on peer influence has looked at screen time in general, which included television watching, video gaming and internet usage [26,27,28]. Fletcher [26] used nationally-representative survey of U.S. adolescents in grades 7 through 12 in 1996 and found that an increase in average school-level television viewing by one hour was associated with an almost half an hour increase in television viewing by an adolescent. Liu et al. [27] investigated involvement in screen activities by U.S. adolescents in grades 7–12 in 1994 using an aggregate of hours a week that students watched television, videos or played video or computer games but did not find a significant peer effect of nominated friends on an adolescent. Sirard et al. [28] used a cross sectional study (n = 2126) in 20 middle schools and high schools in Minneapolis/St. Paul MN during the 2009–2010 academic year and found that every additional hour of a best female friend’s screen time (watching TV/DVDs/videos, using a computer not for homework, and Xbox/Play-Station/other electronic games played when sitting) was associated with an additional 15 minutes of screen time per week for high school boys. While general screen time includes video gaming, a large proportion of it is comprised of television viewing. An important difference between television viewing and video gaming is the level of involvement and the type of activity: Unlike television watching, video games provide a place for social interaction both online and face-to-face, and the content of video games can be modified by interaction of player with the game and with other players. Only one recent study [29] focused on video gaming and found a statistically significant peer effect in console use time in a representative survey of secondary school students aged 14–16 in Catalonia (Spain) in 2008, with an additional hour increase in console gaming by nominated friends within the classroom leading to an increase in individual’s gaming by 5 minutes among boys but no effect among girls.

The existing research into peer effects in media consumption (including video gaming) has several flaws and shortcomings, which we attempt to address in this study. Most importantly, most studies used outdated data, convenience samples or failed to address statistical issues surrounding the estimation of peer effects. For example, samples from 1996 and 1995 were used to look at television viewing and general screen activities [26,27]. However, the development of 128-bit gaming systems (also known as 6^th^ generation systems, including Sony PlayStation 2, Nintendo GameCube, and Microsoft Xbox) in 1998 allowed for development of much more realistic video games, which could change the nature of the experience. Furthermore, this new technology could have potentially changed the type of audience playing these games and the nature of social interactions, as these devices saw a significant rise in the popularity of large-scale online gaming, namely with the introduction of Microsoft’s Xbox Live and Sony’s PlayStation Network online gaming platforms in 2002 and 2006 respectively. Today, these platforms provide a unified gaming experience and cater to millions of console, PC and even smartphone gamers. It seems likely that such platforms make online gaming much more accessible and appealing to a wide audience, while previously online gaming was more restricted to tech-savvy PC gamers. These innovations coincided with the rapid increase in popularity of video gaming since late 1990’s; only four out of the ten best-selling video game consoles in history were made before the year 2000 (http://www.statista.com/statistics/268966/total-number-of-game-consoles-sold-worldwide-by-console-type/ accessed on June 16, 2014). Even today, video gaming constantly evolves into a more realistic and immersive experience, yet most studies on media consumption were conducted using data that preceded this expansion. Another important aspect of modern video games and consoles is that many of them can be played on-line with friends and even strangers and do not require physical presence of the participants–which is in contrast to the previous generations in arcades that were mainly played only in social spheres or together in the sofa.

While some of the existing studies rely on more recent data, the data used were quite limited in coverage: Sirard et al. [28], who studied peer effects in general screen time, only used data collected in the state of Minnesota; Escardíbul et al. [29], who studied peer effects in video gaming time, used only data from Catalonia, an autonomous community of Spain. The results from these limited samples might not directly apply to other populations in light of a significant variability across human populations in terms of psychology, motivation, and behavior [30].

There are also methodological issues that arise when estimating behavioral peer effects. As noted by Manski [31], the identification of (endogenous) peer effects is difficult because of the “reflection problem”—the fact that individuals tend to select their peers, may face similar environment or select each other based on common traits or interests (correlated effects), or may be influenced by peers’ background characteristics (contextual effects). If these are the main driving forces behind the correlation in peers’ behavior, a policy aimed at changing video gaming habits will not have a ripple effect among individuals who were not directly affected by the policy. The previous analysis of peer effects [29] may suffer from the selection concerns; while they utilized the relevant peer group of nominated friends in the same classroom, estimates based on the peer group of nominated friends within the same classroom are likely suffer from peer selection [31] that might not be properly controlled for by the included observable covariates at the individual and classroom levels. Escardíbul et al. [29] tried to address the reflection problem by using a convincing identification strategy—by utilizing characteristics of the respondents’ friends-of-friends who are not friends with the respondent—as instruments for peers’ video gaming. However, the intransitivity property of the peer network, which requires that some of the students are not directly related as friends but only via others, is unlikely to hold if some students under-report their friendships.

Our updated analysis relies on a recent nationally representative sample of the U.S. school-aged adolescents in 2009–2010 school year. Our empirical methods account for the problems of contextual effects, correlated effects, and peer selection and thereby reduce the potential biases from the estimates of peer effects. Our peer group is comprised of non-nominated peers–older adolescents in the same grade in the school—who are unlikely to be selected by the individual yet are likely to exert significant influence on video gaming behavior of an adolescent. We also use instrumental variable strategy where we use measures of peer’s parental control and relationship as instruments. Our analysis also accounts for a number of individual and family-level covariates, which were identified in the literature as important determinants of video gaming behavior and the omission of which may bias the estimates of the peer effects. For example, video gaming has been found to vary by gender, race and ethnicity, body weight, urban status, grade level in school, school performance, and socio-economic status of the household [32,33,34,28,26,35]. Finally, given the evidence that video gaming is much more prevalent among males [36,37,38,39,40] and that peer effect in console use varies by gender [29], we explore whether the peer effect in video gaming time differs by gender. Thus, we propose the following two hypotheses:

1. There is a significant peer effect in adolescents’ video gaming time.
2. Peer effect in video gaming time is stronger among males compared to females.

## Data

We utilize data from Health Behavior in School-Aged Children (HBSC), which is a school-based study of health-related attitudes and behaviors based on a series of cross-sectional national surveys of school-aged children. We use HBSC 2009–2010 data for the U.S. [41], which contain information from the 2009–2010 school year on 12,642 students from 314 public, Catholic, and other private schools and who were enrolled in grades 5, 6, 7, 8, 9, and 10 or their equivalent in the 50 states and the District of Columbia. In most schools in the U.S., education is divided into three levels: elementary school (kindergarten to 5^th^ grade and ages 5–10), middle or junior high school (grades 6^th^ to 8^th^ and ages 11–13), and high school (grades 9^th^ to 12^th^ and ages 14–18). In addition, education is mandatory starting between the ages of five and eight and ending between ages sixteen and eighteen, depending on the state (“US School System.” http://www.fulbright.org.uk/, accessed on 06.04.2016). HBSC 2009–2010 data are maintained and distributed to the general public free of charge (upon registration and agreement with the terms of use) by the Inter-university Consortium for Political and Social Research. (Access and data download page for HBSC 2009–2010 is http://www.icpsr.umich.edu/icpsrweb/ICPSR/studies/34792#accessNotes, accessed on 06.08.2016). The objectives of HBSC are to monitor health-risk behaviors of adolescents in order to identify targets for health promotion initiatives and to help explain the development of health attitudes and behaviors through early adolescence. HBSC contains extensive information on substance use (e.g., tobacco, alcohol, and marijuana) and various health behaviors and attitudes (including video gaming, eating habits, physical activity, body image, health problems) in addition to demographic and socio-economic conditions of the student’s family. (Detailed description of HBSC 2009–2010 can be found at http://www.icpsr.umich.edu/icpsrweb/ICPSR/studies/34792, accessed on 06.08.2016). Students were also asked about their relationship with their parents and the level of parental control over their activities. These questions are used for creating instrumental variables (IV), which are used to help identify the peer effects. Because HBSC surveyed multiple students in the same school and grade, we were able to create same-grade peer group measure of video gaming.

### Measure of Adolescent Video Gaming

The dependent variable of our analysis is a continuous variable measuring the number of hours a day that student spent video gaming and is based on the survey questions “About how many hours a day do you usually play games on a computer or games console (Playstation, Xbox, GameCube, etc.) in your free time on weekdays/weekends?” The reported number of hours played on weekdays multiplied by 5 was added to the number of hours played on weekends multiplied by 2 and the sum was divided by 7 to create a weighted average number of hours spent video gaming by the adolescent during a typical week.

### Peer Measure of Video Gaming

Our peer measure of video gaming is based on co-location and is the average number of hours spent video gaming among students who are one year older than the respondent and belong to the same grade level in the same school as the responder.

Previous studies of peer effects often used nominated peers [28,29] to define peer groups. These groups usually include close friends that were nominated by the student. This appears to be a reasonable approach to defining the relevant peer group, as students are likely to be engaged in discussions about gaming with their friends; they may also participate in gaming together. However, there are several methodological difficulties when trying to identify peer effects among nominated friends. Friends, who are frequently selected by the individual, are likely to share similar background, interests and personalities. Thus, it is difficult to separate the correlated effects from the endogenous peer effect. This is less of a problem when using grade-level peers. While the degree of peer influence of grade-level peers is likely less than that of close friends, classmates and same-grade individuals were previously found to have a significant influence on various behaviors of adolescents [42,43,26,44]. Students often communicate and are exposed to gaming by other students in the same grade, and using grade-level peers will allow us to capture the gaming culture and social norms pertaining to gaming within a grade level.

An advantage of using grade-level peers is that they are assigned exogenously (based on age) and independently of other individual’s characteristics that might be correlated with his/her video-gaming behavior. As a result, the estimated peer effects are less likely to capture correlated effects. In addition, because our grade-level peer measures are based on peers’ own reports, they do not to suffer from the reporting bias from using respondents’ own reports of peers’ activities [45,46]. Friendship nominations, on the other hand, are likely to suffer from recall bias when respondents are presented with a long roster [47]. Using older grade-mates as a peer group also means that individual’s own outcome is not present in the peer group measure (peer group average). This allows to avoid the downward bias in the peer effect estimate, which usually results from the mechanical negative correlation between the individual’s and the peer group’s outcome after systematically removing individual’s own outcome from the peer group’s outcome. This is sometimes referred to as “regression to the mean” in the peer effects literature [48]. If a students’ hours video gaming are subtracted from calculating grade average hours, the grade average hours will be negatively correlated with individual students’ hours: For a student whose hours exceed grade average, grade average is below the student’s hours, and vice versa.

### Family and Personal Characteristics

Our multivariate analysis includes several important individual- and family-level determinants of video gaming among adolescents. Individual characteristics include sex, race (white, black, other race), Hispanic ethnicity, whether student is U.S. born, student’s body mass index (BMI), whether student has ever smoked, whether student is a current drinker, whether student used drugs in the past year and whether student resides in an urban area. Other individual characteristics include a categorical measure of student-reported teacher’s evaluation of student’s performance on school tests (1–4, 1 for ‘Below average,’ 2 for ‘Average,’ 3 for ‘Good,’ and 4 for ‘Very good’) and a categorical measure of student’s feeling about school (1–4, 1 for ‘I don't like it at all,’ 2 for ‘I don't like it very much,’ 3 for ‘I like it a bit,’ and 4 for ‘I like it a lot’). We also use indicators for student’s school and grade level (grade 5 through grade 10 with grade 5 being an omitted category). Family-level variables include indicators of whether the student’s father and mother have a job, whether he or she has siblings, whether mother and father are at home, whether there is a second home, number of computers at home, and a categorical affluence scale measuring how wealthy the family is (1–9, 1 for ‘Low’, 9 for ‘High’).

We also use measures of parental control and relationship with the parent. Parental control and monitoring of adolescent’s activities appears to be an important determinant of adolescent’s media use [49], while lack of significant relationship with parent or lack of mutual attachment are important predictors of early onset of many risky behaviors [50,51,52]. The measures used in our analysis are based on six relevant questions answered by the student on a scale 1–3 (1 for ‘almost always,’ 2 for ‘sometimes,’ and 3 for ‘never’): “Whether parent (or guardian) tries to control everything I do”; “Whether parent (or guardian) understands my problems”; “Whether parent (or guardian) helps me as much as I need”; “Whether parent (or guardian) lets me make my own decisions”; “Whether parent (or guardian) is loving”; and “Whether parent (or guardian) treats me like a baby”. The response ‘almost always’ to each question was coded as one in order to create the corresponding binary indicator variables: “Parent tries to control everything I do” (0 or 1), “Parent understands my problems” (0 or 1), “Parent helps me as much as I need” (0 or 1), “Parent lets me make own decisions” (0 or 1), “Parent is loving” (0 or 1) and “Parent treats me like a baby” (0 or 1). These indicators of parental control and relationship also formed the basis of our instrumental variable identification strategy for estimating peer effects.

The full sample used in the analysis contains 11,888 observations with non-missing values on all variables. In order to reduce measurement error in the estimates of peer group averages, we only used peer group averages that were based on at least two observations. As a result, we have 7,978 non-missing observations for the peer measure. Table 1 reports descriptive statistics for the variables used in our analysis.

**Table 1:** Descriptive statistics (N = 7,978).

Variable	Mean (SD)/Number (%)
*Dependent variable*	
Hours per day gaming, mean (SD)	1.213 (1.351)
*Peer measure (avgerage among older grade mates)*	
Hours per day gaming, mean (SD)	1.275 (0.724)
*Gender*	
Female, n (%)	4117 (51.6%)
Male, n (%)	3861 (48.4%)
*Race*	
White, n (%)	4045 (50.7%)
Black, n (%)	1308 (16.4%)
Other race, n (%)	2625 (32.9%)
*Ethnicity*	
Non-Hispanic, n (%)	5816 (72.9%)
Hispanic, n (%)	2162 (27.1%)
*Other individual-level controls*	
US born, n (%)	7061 (88.5%)
BMI, mean (SD)	21.16 (4.107)
Ever smoked, n (%)	1101 (13.8%)
Current drinker, n (%)	2338 (29.3%)
Used drugs past year, n (%)	144 (1.8%)
Urban residence, n (%)	2377 (29.8%)
Gets good grades (teacher evaluation, 1–4), mean (SD)	3.003 (0.830)
Likes school (1–4), mean (SD)	3.028 (0.850)
*Family-level controls*	
Father has a job, n (%)	6175 (77.4%)
Mother has a job, n (%)	5585 (70%)
Has siblings, n (%)	7172 (89.9%)
Mother at home, n (%)	7204 (90.3%)
Father at home, n (%)	5154 (64.6%)
Has second home, n (%)	5130 (64.3%)
Number of computers, mean (SD)	1.863 (0.913)
Family well off (1–9), mean (SD)	5.979 (1.905)
*Relationship with parent (or guardian)*, *n (%)*	
Parent tries to control everything I do	1213 (15.2%)
Parent understands my problems	2481 (31.1%)
Parent helps me as much as I need	3415 (42.8%)
Parents lets me make own decisions	2138 (26.8%)
Parent is loving	4316 (54.1%)
Parent treats me like a baby	758 (9.5%)
*Grade level*, *n (%)*	
Grade 5	1029 (12.9%)
Grade 6	1396 (17.5%)
Grade 7	1508 (18.9%)
Grade 8	1572 (19.7%)
Grade 9	1316 (16.5%)
Grade 10	1157 (14.5%)
*Instrumental variables (peer average)*, *n (%)*	
Parent tries to control everything I do	1261 (15.8%)
Parent is loving	2401 (30.1%)
Parent helps me as much as I need	3239 (40.6%)
Parents lets make own decisions	2154 (27%)
Parent is loving	4157 (52.1%)
Parent treats me like a baby	774 (9.7%)

## Estimation Strategy

Manski [31] described empirical problems that arise when trying to estimate peer effects using observational data by collectively referring to them as the “reflection problem.” Specifically, the coefficient on the average peer outcome in a standard linear regression may have several interpretations, which may not necessarily reflect behavioral response of an individual to social influence of his or her peers. Manski [31] pointed out three different interpretations for the coefficient on the average peer outcome:

- Endogenous effect–individual’s behavioral response to changes in the behavior of others in his or her peer group. This effect is present if video gaming among peers creates a social norm and social acceptance of gaming and thus leads individual to increase his or her time playing video games. Here, the behavior of peers leads to a change in behavior of an individual, whose behavior, in turn, becomes part of peer group’s behavior. The policy significance of the existence of such a behavioral effect is that targeting some individuals in a group would affect the behavior of other individuals in the group.
- Exogenous (contextual) effect–effect of exogenous (background) characteristics of the peers on individual’s behavior. For example, if most peers come from households with permissive parenting style which allowed peers to spend long hours gaming, individual’s own gaming could also be high as a result. While exogenous effect is also a type of social influence, targeting the behavior of peers will not lead to the same effect as in the case of endogenous effect and the behavior of others in the peer group will not change.
- Correlated effect–describes similarities in behavior of individuals who share the same environment (e.g., geographical, institutional). For example, students in urban schools could all have greater attachment to electronic gadgets compared to students from rural schools. Correlated effect also describes similarities in behavior of individuals who sort themselves into groups based on personal traits. For example, students who have preference for sedentary activities may be more likely to become friends. Again, if one of these students starts spending less time video gaming as a result of an intervention, this change in behavior is not going to spill over to his friends because something else is driving the correlated effects in the first place.

Given these alternative interpretations of a significant peer effect, standard linear regression is unable to distinguish between the endogenous, the exogenous and the correlated effects and a successful policy aimed at changing gaming behaviors will depend upon the mechanism driving peer effect in each particular case. If the purpose is devising an effective policy that would exploit behavioral influences among the peers, the econometric strategy needs to control for all other influences in order to identify the endogenous peer effect [53]. Our empirical strategy deals with the reflection problem by relying on the instrumental variable approach, school fixed effects and by using exogenously assigned peer group.

Because approximately 18 percent of the students in our sample (1,449 observations) do not spend any time playing video games, the dependent variable (hours spent video gaming by a student) is left-censored at zero. In order to account for the censoring, we employ the Tobit regression model, which models the outcomes of the latent variable y* related to the observed values of variable *y* as follows:

y={0ify*≤0y*ify*>0}

$$y_{ijs}^{*}=\beta \prime y_{js}+\gamma \prime X_{ijs}+\delta \prime S_{s}+\varepsilon_{ijs}$$ (1)

In this model, individual hours of video gaming by student *i* belonging to peer group *j* in school *s*, $y_{ijs}^{*}$, is a function of average number of hours of video gaming by the individual’s peers, *y*_*js*_. *X*_*ijs*_ is a vector of personal and family demographic and socio-economic characteristics including a set of grade-level dummy variables. *S*_*s*_ is a vector of school indicator variables (school-level fixed effects), which are included in order to control for the confounding factors that all students in the same school are exposed to. These could include any influences on video gaming at the level of school district (e.g. built school environment, availability of school clubs) as well as community-level environmental influences that might change the way students use their time (e.g., crime and poverty level in the neighborhood, presence of exercise facilities). School and grade fixed effects also control for the correlated effects (including selection via sorting into same grade and school) in the estimate of the peer effect. The endogenous peer effect, *β*, captures peer effect on a student’s time spent video gaming. If *β* is estimated to be positive, then policy intervention that alters video gaming behavior of an individual would have an indirect effect on video gaming of other students in the same peer group [31].

If individual’s behavior is a linear function of peer group average as in Eq 1, the parameter *β* is not identified because group behavior is by definition the aggregation of individual behavior [31]. Therefore, in order to identify the direct effect of peers’ behavior on the adolescent’s behavior, we utilize instrumental variable strategy. Instrumental variable approach is commonly used to identify peer effects in various health-related behaviors of adolescents [54,55,25,56]. We obtain IV estimates of the model in Eq 1 using a two-step method [57,58], where an endogeneity-correction term given by the least-squares residual from the first-stage model is added to the censored regression in Eq 1, and then Tobit regression is applied to the model with the correction term estimated jointly with the first-stage model [59].

The key to implementing the IV technique is having access to instrumental variables which satisfy two properties. First, instruments must have a strong effect on the endogenous regressor, which in our case is peer measure of video gaming time. Second, instruments must have no effect on the outcome of interest, individual’s gaming time, other than through their effect on the peer measure. We use six variables that describe peers’ relationship with their parents and the level of parental control over their activities: The percentage of peers whose parent (or guardian) (i) “tries to control everything they do”, (ii)“understands their problems”, (iii) “helps them as much as they need”, (iv) “lets them make their own decisions”, (v) “is loving”; and (vi) “treats them like a baby”. These peer level variables are expected to directly impact peers’ behavior but should otherwise not predict an individual’s behavior. The intuition for the identification using instruments is that, while individuals whose parents are more permissive or less involved are likely to spend more time playing video games, the parenting style of peer’s parents will only affect the individual via peers. The use of instrumental variables in combination with school-level fixed effects, exogenous peer group and important individual-level controls would allow us to obtain more consistent estimates of the peer effects.

## Results

We begin by presenting Tobit regression estimates of the peer effects of video gaming time in Table 2, where the estimates are only adjusted for the effect of individual and family level covariates (no school fixed effects or IV). The estimates are obtained for everyone, and then stratified by gender. (All of the Stata code used to create the variables for our analysis and to produce the estimates in this paper are available from the authors upon request.)

**Table 2:** Estimates of peer effects in video gaming time (hours/week) using Tobit regression.

	Model for everyone		Model for males		Model for females	
Video gaming time (hours/week)	B (95%CI)	p-value	B (95%CI)	p-value	B (95%CI)	p-value
Hours per day gaming (peer average)	0.095 (0.047, 0.143)	0.000	0.125 (0.057, 0.193)	0.000	0.065 (-0.001, 0.132)	0.053
Male	0.884 (0.816, 0.952)	0.000				
Black	0.423 (0.323, 0.522)	0.000	0.383 (0.242, 0.525)	0.000	0.457 (0.317, 0.596)	0.000
Other race	0.246 (0.153, 0.339)	0.000	0.224 (0.087, 0.360)	0.001	0.257 (0.131, 0.384)	0.000
Hispanic	0.048 (-0.047, 0.144)	0.321	0.050 (-0.089, 0.189)	0.484	0.074 (-0.057, 0.204)	0.268
US born	0.148 (0.036, 0.261)	0.010	0.221 (0.064, 0.377)	0.006	0.053 (-0.109, 0.215)	0.520
BMI	0.010 (0.001, 0.018)	0.021	0.004 (-0.008, 0.017)	0.470	0.015 (0.003, 0.027)	0.012
Ever smoked	-0.034 (-0.146, 0.077)	0.546	-0.101 (-0.254, 0.052)	0.195	0.033 (-0.131, 0.196)	0.693
Current drinker	-0.013 (-0.097, 0.071)	0.763	0.030 (-0.089, 0.149)	0.624	-0.046 (-0.166, 0.073)	0.445
Used drugs past year	0.032 (-0.242, 0.306)	0.820	-0.257 (-0.632, 0.118)	0.179	0.316 (-0.083, 0.715)	0.121
Urban residence	0.168 (0.093, 0.243)	0.000	0.157 (0.049, 0.264)	0.004	0.167 (0.062, 0.272)	0.002
Gets good grades (teacher evaluation, 1–4)	-0.043 (-0.087, 0.000)	0.052	-0.037 (-0.099, 0.025)	0.240	-0.050 (-0.111, 0.011)	0.108
Likes school (1–4)	-0.096 (-0.138, -0.053)	0.000	-0.132 (-0.192, -0.072)	0.000	-0.061 (-0.121, -0.001)	0.045
Father has a job	-0.095 (-0.184, -0.005)	0.039	-0.156 (-0.285, -0.027)	0.018	-0.057 (-0.181, 0.067)	0.368
Mother has a job	-0.071 (-0.147, 0.006)	0.070	0.009 (-0.101, 0.119)	0.875	-0.163 (-0.268, -0.057)	0.002
Has siblings	-0.081 (-0.193, 0.032)	0.159	-0.113 (-0.272, 0.046)	0.163	-0.030 (-0.188, 0.127)	0.705
Mother at home	0.096 (-0.022, 0.213)	0.112	0.145 (-0.017, 0.307)	0.080	0.057 (-0.113, 0.228)	0.510
Father at home	-0.012 (-0.097, 0.073)	0.786	0.002 (-0.118, 0.121)	0.979	-0.014 (-0.134, 0.106)	0.818
Has second home	-0.090 (-0.169, -0.010)	0.028	-0.104 (-0.216, 0.008)	0.068	-0.077 (-0.191, 0.036)	0.180
Number of computers	0.186 (0.135, 0.237)	0.000	0.199 (0.126, 0.272)	0.000	0.170 (0.099, 0.241)	0.000
Family well off (1–9)	-0.021 (-0.046, 0.004)	0.107	-0.030 (-0.066, 0.006)	0.102	-0.012 (-0.047, 0.023)	0.515
Parent tries to control everything I do	0.061 (-0.053, 0.175)	0.297	0.010 (-0.156, 0.175)	0.908	0.124 (-0.034, 0.281)	0.123
Parent understands my problems	0.039 (-0.060, 0.138)	0.437	0.030 (-0.112, 0.173)	0.678	0.032 (-0.105, 0.169)	0.650
Parent helps me as much as I need	-0.149 (-0.254, -0.044)	0.005	-0.179 (-0.329, -0.028)	0.020	-0.124 (-0.269, 0.022)	0.096
Parents lets me make own decisions	0.037 (-0.053, 0.127)	0.419	0.044 (-0.083, 0.172)	0.496	0.028 (-0.099, 0.154)	0.669
Parent is loving	-0.039 (-0.157, 0.078)	0.510	-0.000 (-0.168, 0.167)	0.996	-0.064 (-0.227, 0.100)	0.445
Parent treats me like a baby	0.052 (-0.084, 0.188)	0.450	0.108 (-0.093, 0.310)	0.291	0.021 (-0.162, 0.204)	0.824
Grade 6	0.041 (-0.082, 0.163)	0.515	0.052 (-0.126, 0.229)	0.567	0.019 (-0.148, 0.187)	0.819
Grade 7	0.022 (-0.130, 0.174)	0.777	-0.027 (-0.242, 0.188)	0.806	0.053 (-0.161, 0.266)	0.629
Grade 8	-0.047 (-0.200, 0.106)	0.548	0.109 (-0.103, 0.321)	0.313	-0.227 (-0.448, -0.007)	0.043
Grade 9	-0.251 (-0.412, -0.090)	0.002	-0.050 (-0.277, 0.177)	0.666	-0.457 (-0.686, -0.227)	0.000
Grade 10	-0.345 (-0.515, -0.175)	0.000	-0.019 (-0.259, 0.220)	0.873	-0.694 (-0.935, -0.453)	0.000
Constant	0.036 (-0.215, 0.287)	0.779	0.202 (-0.151, 0.555)	0.262	-0.133 (-0.489, 0.223)	0.464
Observations	7978		3865		4113	
Model chi-squared	1054.743		197.856		296.106	
Model p-value	0.000		0.000		0.000	

Note: B is the coefficient estimate; 95%CI is the 95% confidence interval.

The naïve estimates in Table 2 show that there is a positive and statistically significant association between peers’ and individual’s video gaming time. Among included covariates, male students spend significantly more time video gaming compared to females. Students of black or other race play significantly more compared to students of white race. Higher BMI, urban residence, and number of computers at home are also associated with significantly higher time video gaming. On the other hand, better progress at school, positive attitude towards school, having a father or mother with a job, having a second home, being in the ninth or tenth grade (relative to the fifth grade) and having a helping parent are negatively associated with the time spent video gaming. After stratifying the estimates by gender, video gaming time among males is strongly associated with peers’ video gaming time while the association for females is only marginally statistically significant.

We report estimates of instrumental variable Tobit regression with school fixed effects in Table 3. The estimates on the control variables are not significantly different. We report two tests of our instruments: the F test of the strength of the excluded instruments, and the test for overidentifying restrictions (test of exogeneity of instruments). The F test statistic for the excluded instruments is far greater than 10 in all cases, which indicates that our instruments are strong. The Amemiya-Lee-Newey overidentifying restrictions test fails to reject the null hypothesis that all of our instruments are exogenous (i.e. uncorrelated with the error term in the Tobit regression) in all cases. These results support the validity of our instruments.

**Table 3:** Estimates of peer effects in video gaming time using instrumental variable Tobit regression with school fixed effects. Estimates for everyone and by gender.

	Model for everyone		Model for males		Model for females	
Video gaming time (hours/week)	B (95%CI)	p-value	B (95%CI)	p-value	B (95%CI)	p-value
Hours per day gaming (peer average)	0.185 (-0.141, 0.511)	0.266	0.472 (0.004, 0.939)	0.048	-0.182 (-0.653, 0.289)	0.450
Male	0.883 (0.815, 0.951)	0.000				
Black	0.176 (0.054, 0.298)	0.005	0.124 (-0.055, 0.304)	0.173	0.194 (0.026, 0.362)	0.024
Other race	0.196 (0.097, 0.295)	0.000	0.170 (0.023, 0.317)	0.024	0.207 (0.072, 0.342)	0.003
Hispanic	-0.007 (-0.113, 0.099)	0.895	0.007 (-0.148, 0.163)	0.928	0.005 (-0.141, 0.151)	0.944
US born	0.207 (0.093, 0.321)	0.000	0.286 (0.126, 0.446)	0.000	0.118 (-0.046, 0.281)	0.159
BMI	0.009 (0.000, 0.017)	0.049	0.003 (-0.009, 0.015)	0.621	0.012 (0.001, 0.024)	0.040
Ever smoked	-0.030 (-0.142, 0.083)	0.605	-0.074 (-0.232, 0.083)	0.356	0.006 (-0.158, 0.171)	0.940
Current drinker	-0.019 (-0.104, 0.066)	0.660	0.014 (-0.107, 0.136)	0.819	-0.038 (-0.158, 0.082)	0.537
Used drugs past year	0.052 (-0.224, 0.328)	0.711	-0.166 (-0.550, 0.218)	0.396	0.360 (-0.043, 0.763)	0.080
Urban residence	1.295 (-0.225, 2.815)	0.095	0.538 (-2.528, 3.604)	0.731	-0.022 (-3.153, 3.110)	0.989
Gets good grades (teacher evaluation, 1–4)	-0.050 (-0.093, -0.006)	0.025	-0.047 (-0.109, 0.016)	0.146	-0.053 (-0.114, 0.008)	0.090
Likes school (1–4)	-0.086 (-0.129, -0.043)	0.000	-0.113 (-0.174, -0.052)	0.000	-0.059 (-0.120, 0.002)	0.058
Father has a job	-0.066 (-0.157, 0.025)	0.153	-0.147 (-0.280, -0.013)	0.031	-0.029 (-0.154, 0.097)	0.655
Mother has a job	-0.061 (-0.138, 0.016)	0.119	0.016 (-0.096, 0.129)	0.776	-0.151 (-0.258, -0.044)	0.006
Has siblings	-0.090 (-0.203, 0.022)	0.115	-0.051 (-0.213, 0.110)	0.533	-0.101 (-0.259, 0.057)	0.210
Mother at home	0.109 (-0.008, 0.226)	0.069	0.175 (0.011, 0.339)	0.037	0.093 (-0.079, 0.265)	0.288
Father at home	0.010 (-0.075, 0.095)	0.815	-0.017 (-0.141, 0.106)	0.785	0.017 (-0.103, 0.137)	0.781
Has second home	-0.079 (-0.159, 0.001)	0.052	-0.083 (-0.197, 0.030)	0.150	-0.064 (-0.179, 0.050)	0.271
Number of computers	0.177 (0.126, 0.229)	0.000	0.202 (0.127, 0.277)	0.000	0.146 (0.074, 0.219)	0.000
Family well off (1–9)	-0.007 (-0.033, 0.018)	0.569	-0.022 (-0.060, 0.015)	0.238	0.007 (-0.028, 0.043)	0.682
Parent tries to control everything I do	0.062 (-0.051, 0.175)	0.283	0.008 (-0.160, 0.176)	0.927	0.138 (-0.019, 0.295)	0.085
Parent understands my problems	0.032 (-0.066, 0.129)	0.528	0.004 (-0.139, 0.146)	0.959	0.006 (-0.130, 0.142)	0.933
Parent helps me as much as I need	-0.125 (-0.229, -0.020)	0.019	-0.093 (-0.245, 0.059)	0.231	-0.134 (-0.279, 0.012)	0.072
Parents lets me make own decisions	0.024 (-0.065, 0.113)	0.598	0.030 (-0.098, 0.159)	0.643	0.044 (-0.083, 0.170)	0.496
Parent is loving	-0.009 (-0.126, 0.108)	0.882	-0.006 (-0.174, 0.162)	0.944	-0.022 (-0.187, 0.142)	0.791
Parent treats me like a baby	0.064 (-0.071, 0.199)	0.354	0.145 (-0.058, 0.349)	0.162	0.026 (-0.158, 0.209)	0.785
Grade 6	-0.076 (-0.268, 0.116)	0.439	-0.246 (-0.535, 0.043)	0.095	-0.021 (-0.280, 0.237)	0.872
Grade 7	0.158 (-0.369, 0.685)	0.558	-0.026 (-0.801, 0.750)	0.948	0.306 (-0.417, 1.029)	0.407
Grade 8	0.069 (-0.458, 0.596)	0.798	0.017 (-0.756, 0.789)	0.966	0.069 (-0.656, 0.794)	0.851
Grade 9	-0.142 (-0.718, 0.435)	0.630	-0.024 (-0.826, 0.778)	0.954	-0.239 (-1.096, 0.619)	0.586
Grade 10	-0.207 (-0.788, 0.374)	0.485	-0.101 (-0.907, 0.705)	0.806	-0.398 (-1.264, 0.469)	0.368
Constant	-0.106 (-1.652, 1.439)	0.893	1.456 (-1.705, 4.618)	0.367	0.785 (-2.207, 3.778)	0.607
Observations	7978		3865		4113	
Model chi-squared	1620.862		627.575		7168.393	
Model p-value	0.000		0.000		0.000	
Amemiya-Lee-Newey overid Chi-square	2.796		4.998		2.158	
Amemiya-Lee-Newey overid p-value	0.731		0.416		0.827	
F-statistic for excluded instruments	62.256		27.826		30.915	
F-statistic p-value	0.000		0.000		0.000	

Note: B is the coefficient estimate; 95%CI is the 95% confidence interval. All models include school fixed effects.

After stratifying by gender, peer effect is statistically significant only for males and indicates that an increase in the average amount of time playing video games among older grade-mates by an hour leads to .47 hours increase in video gaming by a male student. The peer effect for females is insignificant. These results are consistent with our second hypothesis and partially consistent (for males) with our first hypothesis. In addition, we note that the magnitude of the effect is larger than the corresponding coefficient in Table 2. This indicates that after accounting for the reflection problem, peer effect becomes more important. The estimated peer effect for the entire sample is not significant, which does not lend support to our first hypothesis,

## Conclusion

The objective of this paper is to estimate peer effect in video gaming time among adolescents. We use a nationally representative sample of the U.S. school-aged adolescents surveyed in 2009–2010 to estimate a structural model that accounts for the potential biases in the estimate of the peer effect. Our results suggest that the amount of time spent video gaming by school peers significantly affects own time playing video games among male adolescents; an additional one hour of playing video games per week by peers results in .47 hours increase in video gaming time by male adolescent. We do not find significant peer effect among female adolescents.

Our finding of a significant peer effect in video gaming among males is in line with the earlier research [29], which estimated peer effects in console use among secondary school students aged 14–16 in Catalonia (Spain) using 2008 data. However, the magnitude of the peer effect found in Spanish data is much lower than here; Spanish data [29] showed that an additional hour increase in console use by nominated friends within the classroom leads to an increase in individual’s gaming by only 5 minutes. Our finding of a significant peer effect is also broadly consistent with the literature finding a significant peer effect in general screen time activities, which include video gaming among other media consumption [26,28].

While our paper does not examine the reasons why there is a significant peer effect in video gaming time for males but not females, there are at least two possibilities for this gender difference. One possibility is that there are simply more opportunities for male gamers to interact and influence each other’s gaming habits compared to female gamers, given that the research commonly finds that males play video games much more than females [36,37,38,39,40]. Another possibility is that males and females have different motivations to play video games, with males being more motivated by the opportunity to socialize (and hence influence and being influenced by peers) compared to females. This idea is supported by the finding that boys utilize violent or sports games as social tools that allow for socialization through competition and cooperation [60]. Males also dominate LAN (Local Access Network) gaming events, where players bring their computers to a designated location to play online and face-to-face; and the main gratifying property of LAN events is the opportunity to game in each other’s presence [61]. In addition, in a study of Matese men and women [36] found that “males’ preference for first person shooters, roleplaying games, and sport and strategy games indicates gratification of different needs–challenge and social interaction” ([36], p. 36). On the other hand, women were found to be interested primarily in puzzle, adventure, fighting, and managerial games, which indicates “females’ top reasons for playing include challenge and arousal” ([36], p. 36). Males have also been found to have a higher score on social interaction compared to females in explorative survey (N = 760) conducted among players of *The Sims2* [62].

As with any empirical strategy, our results may only be viewed as suggesting a strong association. Our study points in that direction provided that our assumptions hold. Specifically we assume that our instrumental variables, which reflect parental control over an adolescent’s video-gaming, are plausibly uncorrelated with the video-gaming time of students who are in the same grade. In addition, the data that we use and our estimates only suggest the existence of peer effects in video gaming time, but not the mechanism underlying the process (e.g., observation of behavior vs. participation in behavior). Another limitation of our study is that it uses self-reported time spent playing video games. In fact, it has been found that gamers tend to under-estimate the time they play [63,64]. As long the amount of underreporting of the gaming time is unrelated to the regression error term, our estimates will be attenuated and will provide a lower bound on the magnitude of the (positive) peer effects.

Our findings imply that future policies aimed at changing video gaming behavior among adolescents could potentially benefit from relying on the spread of this behavior among peers, where even the individuals who did not directly participate or were not included in the program could nevertheless be affected by the program. Policies involving only a subset of the target population would have a spillover effect on other individuals and may be more cost effective than previously thought.

Given that video gaming is a global phenomenon, future research could focus on cross-country comparisons to better understand the influence of cultural, ethnic, and other differences on the magnitudes of peer effects in video gaming. It would also be valuable to learn about the mechanisms through which peer effect in video gaming might operate by conducting qualitative analysis of players and by estimating peer effects for different types of games (multiplayer, LAN games, on-line games). Future studies should also confirm whether the gender difference in the peer effect found here exists in other datasets and try to ascertain the reasons behind it.
